# Evaluation of retinal pigment epithelium changes in serous pigment epithelial detachment using synthesized multi-contrast polarization-sensitive optical coherence tomography

**DOI:** 10.1038/s41598-025-09302-6

**Published:** 2025-07-07

**Authors:** Kosei Yanagida, Masahiro Miura, Hidetaka Noma, Toshihiro Mino, Shinnosuke Azuma, Thitiya Seesan, Shuichi Makita, Yoshiaki Yasuno

**Affiliations:** 1 Department of Ophthalmology, Tokyo Medical University, Ibaraki Medical Center, 3-20-1 Chuo, Inashiki, Ami, 300395 Ibaraki Japan; 2 Computational Optics Group, University of Tsukuba, Tsukuba, Japan; 3 Topcon Corporation, Tokyo, Japan

**Keywords:** Polarization-sensitive optical coherence tomography, Convolutional neural network, Retinal pigment epithelium, Melanin, Age-related macular degeneration, Pigment epithelial detachment, Eye diseases, Macular degeneration, Translational research

## Abstract

Retinal pigment epithelium (RPE) melanin thickness maps, derived from multi-contrast images—including the degree of polarization uniformity (DOPU), optical coherence tomography (OCT) angiography, and the attenuation coefficient—are obtained using multi-contrast polarization-sensitive OCT (PS-OCT). These maps have demonstrated utility for three-dimensional assessment of changes in melanin within the RPE (RPE-melanin). While both OCT angiography and the attenuation coefficient can be derived from conventional OCT, measuring the DOPU requires PS-OCT, which is not available on standard commercial OCT systems. To overcome this limitation, we utilized a convolutional neural network to generate DOPU-like images from standard OCT images and used these to calculate a synthesized RPE-melanin thickness map. We evaluated 22 eyes from 20 patients with serous pigment epithelial detachment (PED) secondary to age-related macular degeneration. Both original and synthesized RPE-melanin thickness maps were calculated from multi-contrast PS-OCT datasets. Active RPE lesions were defined as areas with RPE-melanin thickness of ≥ 70 μm (originalRPE_70_ and synRPE_70_ for the original and synthesized maps, respectively). Both synthesized and original RPE-melanin thickness maps closely resembled near-infrared autofluorescence imaging. Furthermore, both the originalRPE_70_ area and synRPE_70_ area were significantly positively correlated with the PED volume. Synthesized RPE-melanin thickness maps may be useful for clinical quantification of RPE-melanin.

**Supplementary Information:**

The online version contains supplementary material available at 10.1038/s41598-025-09302-6.

## Introduction

Age-related macular degeneration (AMD) is a leading cause of vision loss in older adults^1^. Changes in the retinal pigment epithelium (RPE) are a key clinical feature of AMD^2^. Pigment epithelial detachment (PED) is commonly observed in patients with AMD^3^. In eyes with PED—particularly drusenoid and serous types—RPE cells exhibit a range of responses, including sloughing, shedding, hypertrophy, and intraretinal migration^4–6^. These RPE changes are important indicators of RPE function, making their clinical evaluation essential for AMD management^4^.

Clinical retinal autofluorescence (AF) imaging is widely used to assess RPE alterations in AMD^7,8^. Short-wavelength AF (SW-AF), induced by excitation at 488 nm, primarily originates from lipofuscin or melanolipofuscin^9^. Near-infrared AF (NIR-AF), induced by excitation at 785 nm, mainly arises from melanin or melanolipofuscin^10,11^. Simultaneous hyper-AF in both SW-AF and NIR-AF likely indicates increased melanolipofuscin concentration within RPE cells, along with RPE cell thickening and intraretinal migration^6,12^. Despite its widespread use, a significant limitation of clinical AF imaging is the lack of topographical information.

Intensity-based optical coherence tomography (OCT) (i.e., standard OCT) is another crucial clinical tool for evaluating RPE changes^4,5,13^. Some intraretinal hyperreflective foci observed in standard OCT images are thought to result from intraretinal RPE migration^14^, which may also contribute to hyper-AF lesions^6,7,12^. Certain thickened RPE–Bruch’s membrane bands seen in standard OCT may reflect RPE dysmorphia and stacking^2,4^. Although standard OCT provides valuable information about RPE changes, its ability to directly visualize and assess RPE cells is limited by the lack of specific contrast for these cells, hindering direct comparison with AF images.

Polarization-sensitive OCT (PS-OCT) enhances tissue differentiation in retinal diseases^15,16^. Melanin-induced light scattering within the RPE causes depolarization, which is quantified by the degree of polarization uniformity (DOPU)^17,18^. DOPU values range from 0.0 to 1.0, with lower values indicating greater depolarization or polarization scrambling. Combining PS-OCT and AF imaging enables comprehensive assessment of RPE changes in AMD^6,12,19^. However, a key limitation of PS-OCT in evaluating RPE changes is the difficulty in distinguishing between melanin within the RPE (RPE-melanin) and melanin in the choroid^12,19^. To address this, we developed an algorithm using multi-contrast PS-OCT to automatically highlight RPE-melanin^20^. Multi-contrast PS-OCT simultaneously acquires OCT angiography, PS-OCT, and standard OCT images, from which we generated RPE-melanin–specific contrast OCT (RPE-melanin OCT) images for evaluating RPE changes^20^. Both NIR-AF and RPE-melanin OCT imaging are believed to be sensitive to RPE-melanin changes^10,11,20^. Prior work in AMD and Vogt–Koyanagi–Harada disease has shown similarities between NIR-AF and RPE-melanin OCT projection images, suggesting that combining RPE-melanin OCT and AF imaging offers value for quantitatively assessing RPE changes in macular diseases^12,19^.

However, a significant challenge remains in the clinical application of RPE-melanin OCT imaging. This imaging is calculated based on the DOPU, the attenuation coefficient, and OCT angiography. While the attenuation coefficient and OCT angiography can be derived using commonly available commercial retinal OCT devices, calculating the DOPU requires PS-OCT^17^. In PS-OCT, detecting the polarization state of the backscattered probe light requires capturing two orthogonal polarization components. This setup necessitates two detectors and a polarization splitter in addition to the components of a conventional retinal OCT system^16^. As a result, PS-OCT is more complex and costly than standard clinical retinal OCT, limiting its use to research settings. Deriving the DOPU from standard OCT with single-polarization detection would expand the capabilities of existing OCT systems and enable RPE-melanin OCT calculation without the need for PS-OCT.

Deep learning has significantly advanced the field of ophthalmology^21^. Recent research has demonstrated the potential of deep learning to generate PS-OCT images—including metrics such as DOPU and retardation—from standard OCT images^22–24^. Our group employed a convolutional neural network to create DOPU images from standard retinal OCT data^22^. We showed that these synthesized DOPU images are useful for visualizing AMD-related RPE changes, achieving results comparable to those obtained using the DOPU derived directly from PS-OCT^22^. A key advantage of using the synthesized DOPU rather than the PS-OCT-derived DOPU is that it enables the calculation of RPE-melanin OCT imaging from standard OCT data. In this study, we generated synthesized RPE-melanin OCT images using the attenuation coefficient and OCT angiography data combined with synthesized DOPU images. We then evaluated the effectiveness of these synthesized RPE-melanin OCT images for quantifying AMD-related RPE changes and compared the results with established RPE-melanin OCT imaging.

## Methods

### Participants

This prospective, observational, cross-sectional study was conducted in accordance with the Declaration of Helsinki and approved by the Tokyo Medical University Institutional Review Board (T2019-0072 and T2019-0217). It was registered with the University Hospital Medical Information Network (UMIN000039650 and 000039648; http://www.umin.ac.jp/ctr/index-j.htm). All participants provided written informed consent after receiving a full explanation of the study’s nature and implications.

Thirty-six healthy eyes from 36 Japanese participants (8 men, 28 women; age range, 20–61 years; mean age, 39.3 years) were evaluated, with the right eye of each participant selected for analysis. The exclusion criteria were a history of intraocular surgery and any history or evidence of chorioretinal, vitreoretinal, or glaucomatous disease. Additionally, 22 eyes from 20 Japanese patients with serous PEDs due to AMD (15 men, 5 women; age range, 56–84 years; mean age, 71.6 years) were examined. Eyes with retinal diseases other than AMD were excluded. Serous PED was defined as serous elevation of the RPE without retinal hemorrhage, hard exudates, or choroidal neovascularization, the latter of which was ruled out using commercial retinal OCT angiography (DRI-OCT Triton; Topcon, Tokyo, Japan). To avoid treatment-related influences, eyes with prior intravitreal injections, photodynamic therapy, or laser treatment were excluded, as were eyes with severe cataracts or other conditions significantly impairing image quality.

Axial length was measured using an optical biometer (OA-2000; Tomey, Nagoya, Japan). The mean ± standard deviation axial length was 25.1 ± 1.5 mm (range, 22.4–28.0 mm) for healthy eyes and 23.1 ± 1.1 mm (range, 20.5–25.7 mm) for eyes with serous PEDs.

### Multi-contrast PS-OCT

A prototype multi-contrast PS-OCT system was used, as described in previous publications^25,26^. This system simultaneously acquires standard OCT images, OCT angiography, and DOPU data. Standard OCT images were generated by coherently combining four repeated scans. OCT angiography was processed using a noise-corrected, complex-correlation-based method^27^, and DOPU data were calculated using Makita’s noise correction with a 3- × 3-pixel kernel^28^. The system employs a 1.0-µm swept-source laser, achieving an axial scan rate of 100,000 A-scans per second and a 6.0-µm axial resolution in tissue. Volumetric scans covered a 6.0- × 6.0-mm area on the retina using a horizontal-fast raster scanning protocol of 512 A-lines by 256 B-scans. Transverse magnification was calibrated using a modified Littmann’s method for quantitative measurements^29^. Four repeated B-scans were acquired at each location, resulting in a volumetric acquisition time of 6.6 s. Only multi-contrast PS-OCT volumes free of significant motion artifacts were included in the study.

### Synthesized DOPU

The DOPU synthesizing network, based on U-Net^30^, was used to generate the synthesized DOPU images. Details of the network architecture and training process are provided in our previous work^22^. Briefly, the training and validation datasets included 38 normal eyes of 26 healthy volunteers and 457 pathological eyes of 330 patients. The pathological cases included AMD, diabetic retinopathy, central serous chorioretinopathy, uveitis, retinal vein occlusion, and pathologic myopia. None of the participants in the present study were included in either dataset. The dataset, comprising volumetric OCT data, was divided on a subject-wise basis into training and validation sets using an 8:2 ratio. In total, 69,478 patches were used for training and 17,382 patches for validation. The DOPU synthesizing network was trained with standard OCT images as input and the corresponding DOPU images as ground truth, using binary cross-entropy as a cost function. The performance of the method was evaluated on both healthy and pathological eyes. The synthesized DOPU images closely resembled the actual DOPU images, and their ability to visualize RPE abnormalities showed a high level of consistency^22^.

### Synthesized RPE-melanin OCT imaging

Details of the original RPE-melanin OCT imaging have been published previously^20^. To automatically distinguish RPE-melanin from choroidal melanin in DOPU images, an index called F_RPE_ was calculated. This index incorporates the attenuation coefficient^31^, the DOPU, and the blood flow signal from OCT angiography:

$${\text{F}}_{{{\text{RPE}}}} = {\text{ attenuation }}\;{\text{coefficient }} \times \left( {1 - {\text{DOPU}}} \right) \times \left( {1 - {\text{binarized}}\;{\text{ OCT}}\;{\text{ angiography}}\;{\text{ signal}}} \right)$$

**Fig. 1:**
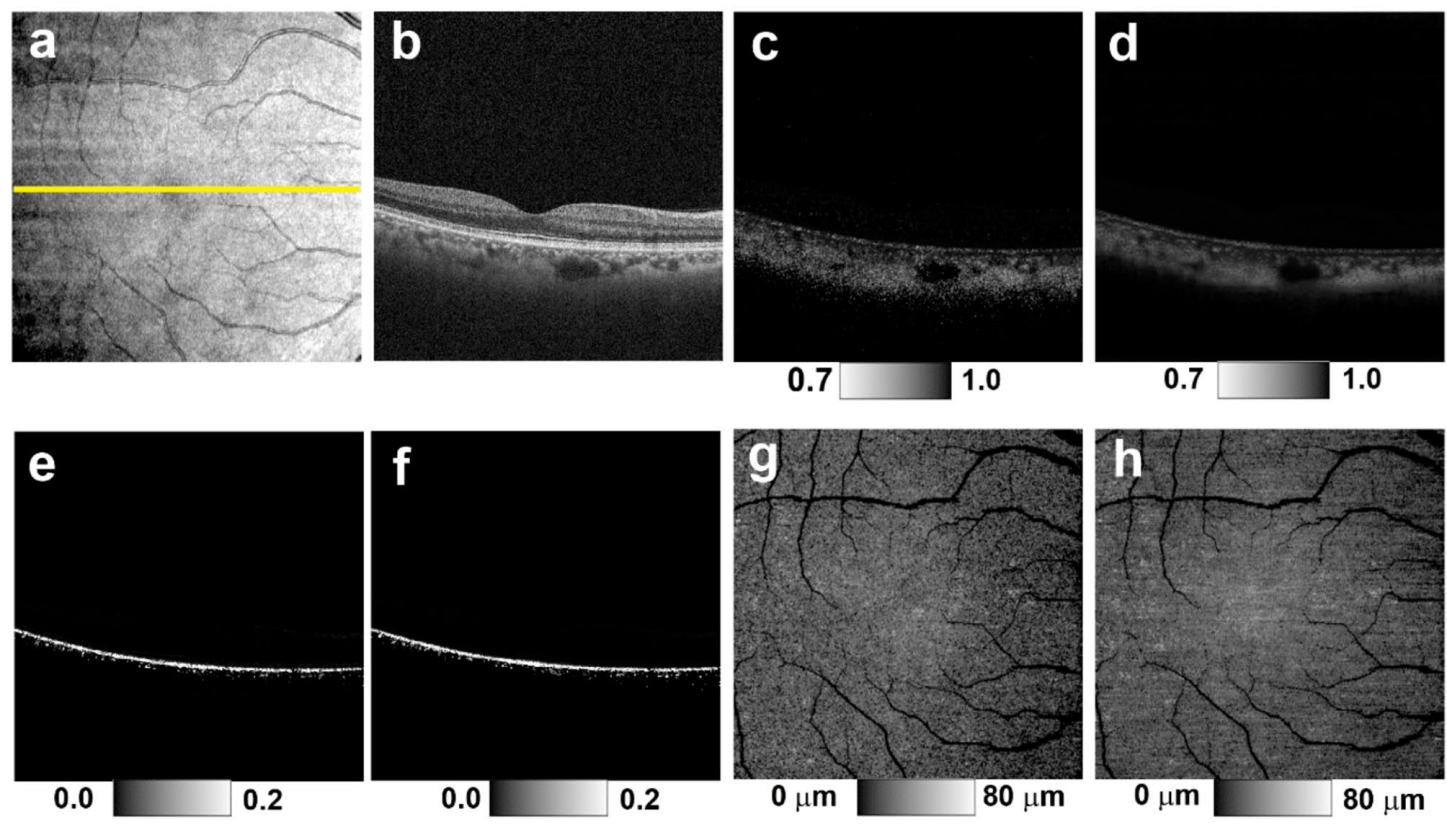
Original and synthesized RPE-melanin OCT imaging of the healthy right eye of a 49-year-old man. (**a**) *En face* projection image of standard OCT. The yellow line indicates the scanning position of the multi-contrast PS-OCT B-scan images shown in (**b**–**f**). (**b**) Standard OCT B-scan image. (**c**) DOPU B-scan image. (**d**) Synthesized DOPU B-scan image. (**e**) Original RPE-melanin B-scan image. (**f**) Synthesized RPE-melanin B-scan image. (**g**) Original RPE-melanin thickness map. (**h**) Synthesized RPE-melanin thickness map.

F_RPE_ was designed to specifically highlight RPE-melanin by leveraging the absence of blood flow within the RPE. A high F_RPE_ value suggests the presence of RPE-melanin. RPE-melanin B-scan images were generated by mapping the F_RPE_ distribution within each B-scan, allowing for assessment of the depth-resolved RPE-melanin distribution (Fig. 1). RPE-melanin thickness maps were then created by counting pixels with high F_RPE_ values (≥ 0.15) along each A-line in the volumetric dataset (Fig. 1).

**Fig. 2:**
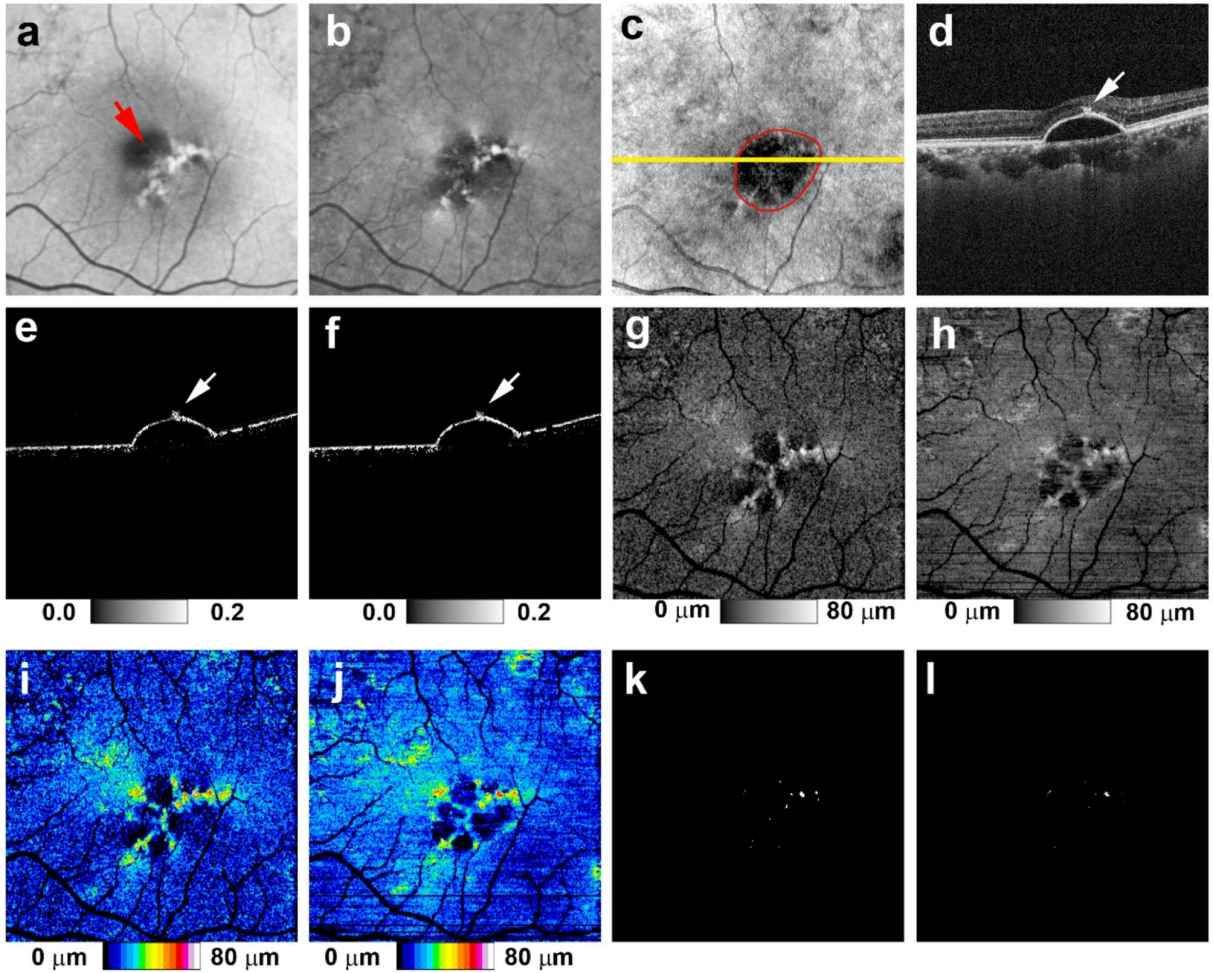
Original and synthesized RPE-melanin OCT imaging of serous PED in the left eye of a 70-year-old man. (**a**) SW-AF and (**b**) NIR-AF images show hyper-AF lesions. (**a**) In the SW-AF image, the hyper-AF lesion is partially obscured in the foveal region (red arrow). (**c**) *En face* projection image of standard OCT. The red line indicates the margin of the PED, and the yellow line indicates the scanning position for B-scan images in (**d**–**f**). (**d**) The standard OCT B-scan image shows a thickened RPE band at the site of the hyper-AF lesion. Both the (**e**) original and (**f**) synthesized RPE-melanin B-scan images show melanin accumulation at the RPE band. The (**g**) original and (**h**) synthesized RPE-melanin thickness maps closely resemble the (**b**) NIR-AF image, with RPE-melanin thickness clearly visualized in (**i**,**j**) the color-coded maps. (**k**) *En face* distribution of originalRPE_70_. (**l**) *En face* distribution of synRPE_70_.

**Fig. 3:**
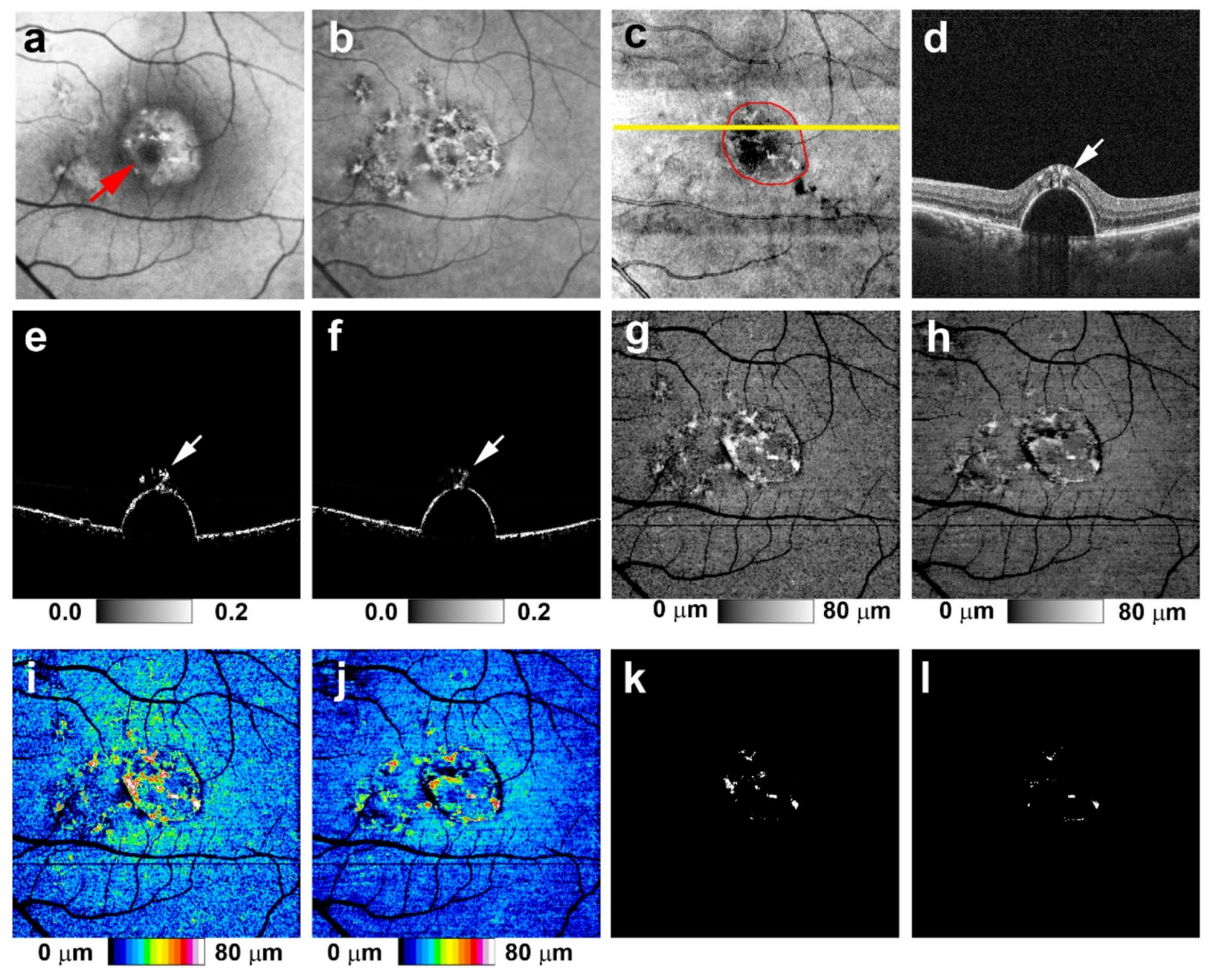
Original and synthesized RPE-melanin OCT imaging of serous PED in the left eye of a 56-year-old man. (**a**) SW-AF and (**b**) NIR-AF images show hyper-AF lesions. (**a**) In the SW-AF image, the hyper-AF lesion is partially obscured in the foveal region (red arrow). (**c**) *En face* projection image of standard OCT. The red line indicates the margin of the PED, and the yellow line indicates the scanning positions for B-scan images in (**d**–**f**). (**d**) The standard OCT B-scan image shows intraretinal hyperreflective foci within the hyper-AF lesion. Both the (**e**) original and (**f**) synthesized RPE-melanin B-scan images reveal intraretinal RPE-melanin migration. The (**g**) original and (**h**) synthesized RPE-melanin thickness maps are similar to the (**b**) NIR-AF image, with RPE-melanin thickness clearly visualized in (**i**,**j**) the color-coded maps. (**k**) *En face* distribution of originalRPE_70_. (**l**) *En face* distribution of synRPE_70_.

Synthesized RPE-melanin OCT images were derived by computing synthesized F_RPE_, which was obtained by substituting synthesized DOPU for DOPU in the F_RPE_ calculation. From this, synthesized RPE-melanin B-scans and thickness maps were generated (Fig. 1). For eyes with serous PED, the area of thickened RPE-melanin (≥ 70 μm: originalRPE_70_ for original, synRPE_70_ for synthesized) was quantified from these maps using Fiji^32^ (Figs. 2 and 3).

### Multimodal imaging

Multimodal imaging was performed to compare both original and synthesized RPE-melanin OCT images with NIR-AF and SW-AF images (Figs. 2 and 3). NIR-AF images (785-nm excitation, emission > 800 nm) and SW-AF images (488-nm excitation, emission > 500 nm) were acquired using an HRA2 system (Heidelberg Engineering, Heidelberg, Germany) and saved as eight-bit grayscale images. Retinal vasculature was manually aligned across the AF and OCT images using Adobe Photoshop version 26.3 (Adobe Systems, San Jose, CA, USA) to facilitate comparison. A retina specialist (M.M.) then subjectively assessed the distribution of hyper-AF lesions.

For eyes with PEDs, the PED volume was calculated by manually segmenting the inner boundary of the PED in each OCT B-scan. These individual segment volumes were then summed using the Cavalieri principle of stereology^33^ to determine the total PED volume.

Statistical analyses were performed using IBM SPSS Statistics for Windows, version 29.0 (IBM Corp., Armonk, NY, USA). Statistical significance was defined as *p* < 0.05.

## Results

**Fig. 4:**
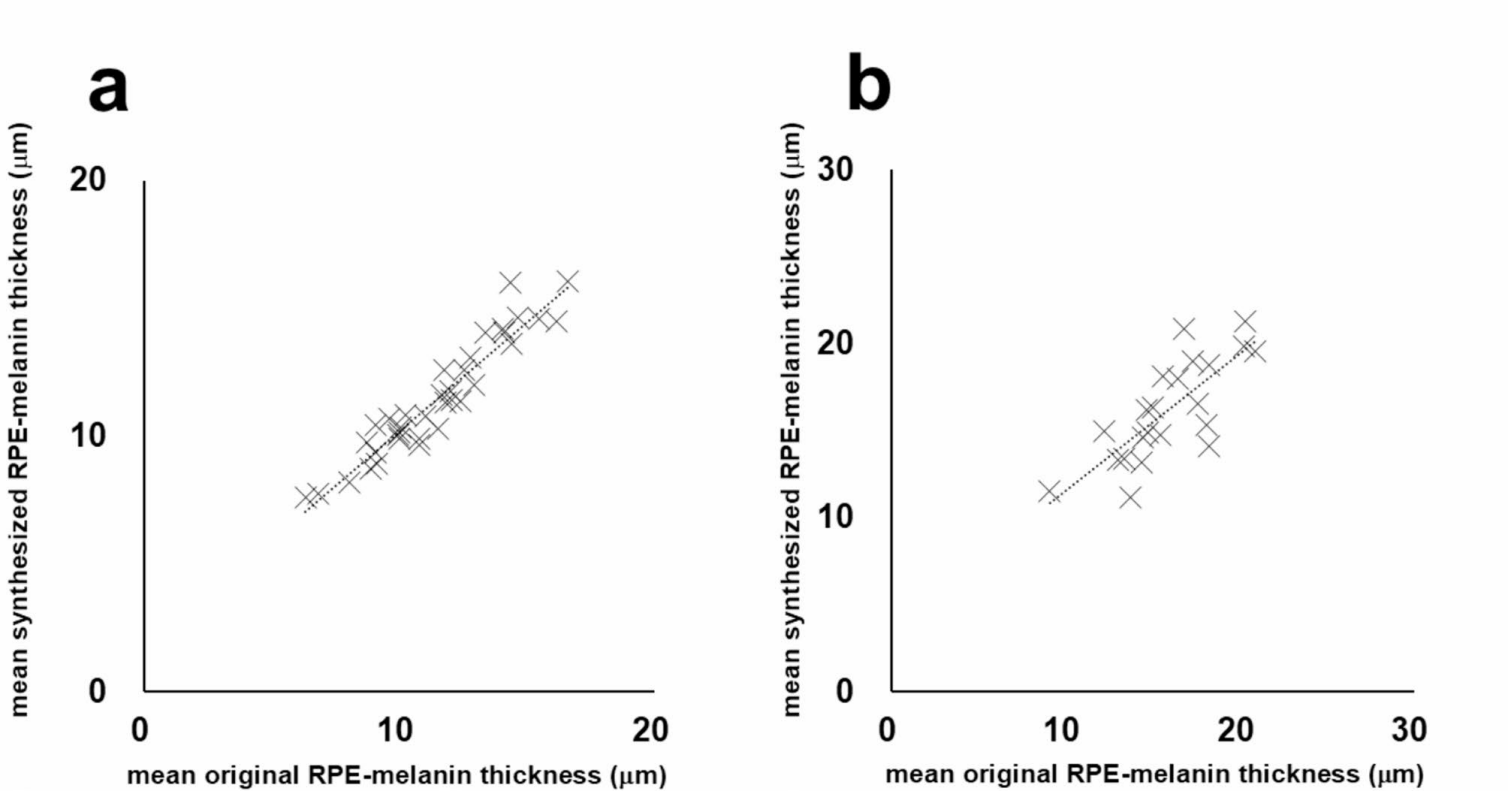
(**a**) Scatterplot showing the correlation between mean original RPE-melanin thickness and mean synthesized RPE-melanin thickness in healthy eyes, with a statistically significant positive correlation. (**b**) Scatterplot showing the correlation between mean original RPE-melanin thickness and mean synthesized RPE-melanin thickness in eyes with serous PEDs, also demonstrating a statistically significant positive correlation.

In healthy eyes, the mean RPE-melanin thickness across the scanning area was 11.5 ± 2.5 μm (range, 6.3–16.6 μm) for the original RPE-melanin thickness map and 11.5 ± 2.3 μm (range, 7.6–16.1 μm) for the synthesized map. There was no significant difference in mean RPE-melanin thickness between the original and synthesized maps (*p* = 0.99, Wilcoxon signed-rank test). The original RPE-melanin thickness map showed a significant positive correlation with the synthesized map in mean RPE-melanin thickness (*p* < 0.0001, Pearson’s correlation coefficient = 0.95) (Fig. 4).

In eyes with serous PEDs, NIR-AF imaging revealed hyper NIR-AF lesions within the PED (Figs. 2 and 3) in 17 of the 22 eyes examined (77%). These lesions corresponded to areas of hyper SW-AF (Figs. 2 and 3); however, hyper SW-AF was partially masked in the foveal region, likely because of macular pigment. The synthesized RPE-melanin thickness maps closely resembled the original RPE-melanin thickness maps. Furthermore, both the original and synthesized thickness maps corresponded well with NIR-AF images in all patients (Figs. 2 and 3). Hyper NIR-AF lesions coincided with areas of thickened RPE-melanin, facilitating quantitative evaluation of RPE-melanin changes in hyper NIR-AF lesions using both original and synthesized thickness maps. The thickness of RPE-melanin within the PED could be easily visualized using the synthesized color-coded RPE-melanin thickness maps, similar to the original maps (Figs. 2 and 3). Synthesized RPE-melanin B-scan images, like the original B-scan images, clearly showed that thickened RPE-melanin lesions were composed of RPE-melanin accumulation at the RPE band and intraretinal RPE-melanin migration (Figs. 2 and 3). In eyes with serous PED, the mean RPE-melanin thickness in the measurement area was 15.9 ± 2.9 μm (range, 9.1–20.9 μm) for the original thickness map and 16.2 ± 2.9 μm (range, 11.6–21.3 μm) for the synthesized map. There was no significant difference in mean RPE-melanin thickness between the original and synthesized thickness maps (*p* = 0.40, Wilcoxon signed-rank test). The original RPE-melanin thickness map showed a significant positive correlation with the synthesized map in mean RPE-melanin thickness (*p* < 0.0001, Pearson’s correlation coefficient = 0.77) (Fig. 4).

**Fig. 5:**
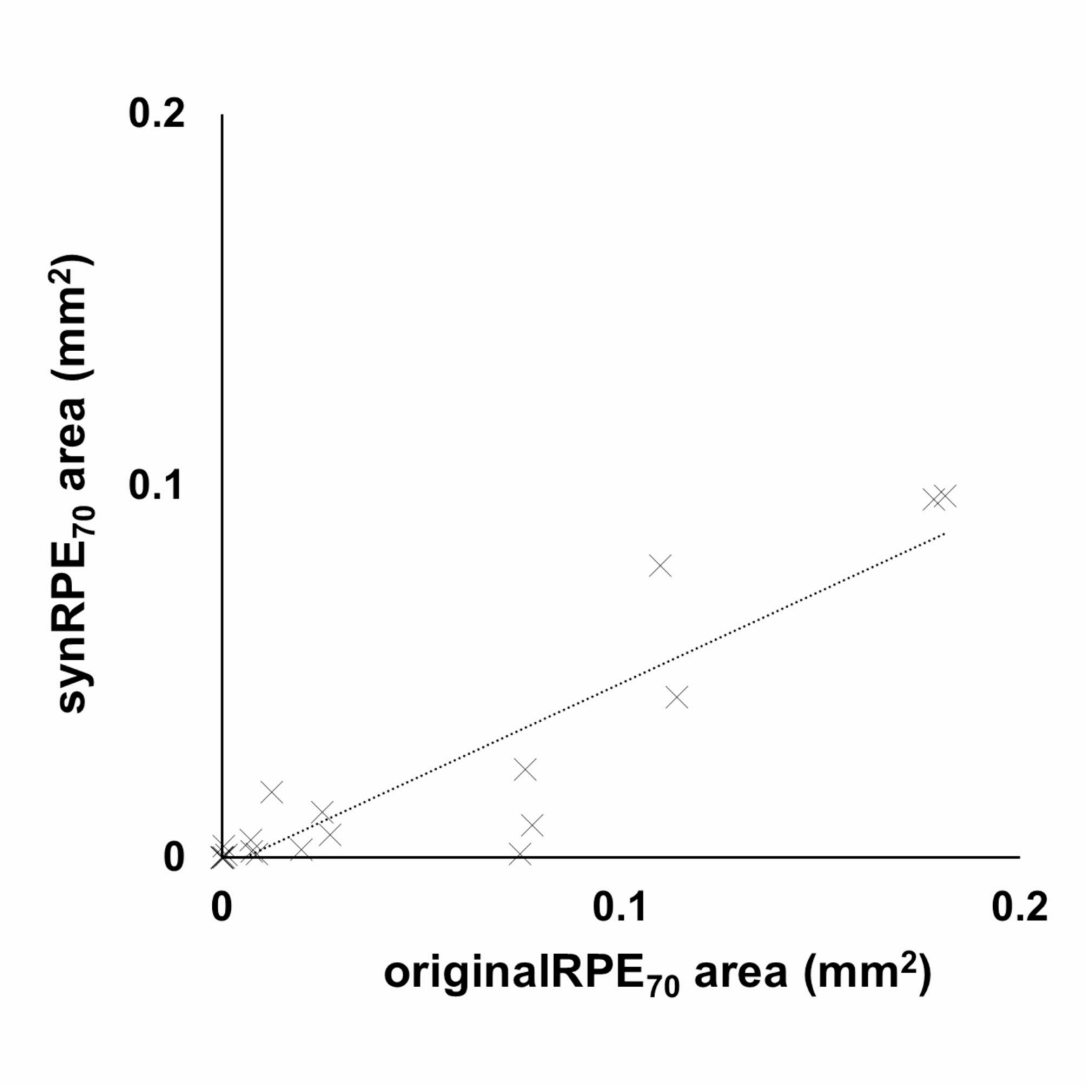
Scatterplot showing the correlation between originalRPE_70_ area and synRPE_70_ area, demonstrating a statistically significant positive correlation.

**Fig. 6:**
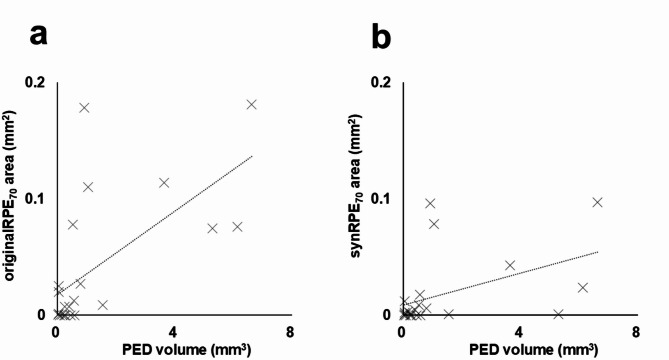
(**a**) Scatterplot showing the correlation between originalRPE_70_ area and PED volume, demonstrating a statistically significant positive correlation. (**b**) Scatterplot showing the correlation between synRPE_70_ area and PED volume, also demonstrating a statistically significant positive correlation.

Next, the area of RPE-melanin thickened lesions (≥ 70 μm) within the PED region was examined using the original and synthesized RPE-melanin thickness maps and compared with the PED volume (Fig. 5). The mean originalRPE_70_ area was 0.042 ± 0.058 mm^2^ (range, 0.000–0.181 mm^2^), and the mean synRPE_70_ area was 0.018 ± 0.031 mm^2^ (range, 0.000–0.097 mm^2^). The mean originalRPE_70_ area was significantly larger than the synRPE_70_ area (*p* = 0.001, Wilcoxon signed-rank test). The synRPE_70_ area showed a significant positive correlation with the originalRPE_70_ area (*p* < 0.001, Pearson’s correlation coefficient = 0.92) (Fig. 5). The mean PED volume was 1.39 ± 2.16 mm^3^ (range, 0.003–6.93 mm^3^). Both the originalRPE_70_ area and synRPE_70_ area showed significant positive correlations with the PED volume (*p* = 0.001, Pearson’s correlation coefficient = 0.64 for originalRPE_70_; *p* = 0.036, Pearson’s correlation coefficient = 0.45 for synRPE_70_) (Fig. 6).

## Discussion

This study compared original and synthesized RPE-melanin OCT imaging in normal eyes and those with serous PED secondary to AMD. Original and synthesized RPE-melanin OCT imaging showed a strong correlation, particularly in normal eyes. In eyes with serous PED, the synthesized RPE-melanin thickness maps closely resembled NIR-AF images, consistent with prior reports using original RPE-melanin thickness maps^12^. This similarity highlights the complementary nature of these imaging techniques. Furthermore, in eyes with serous PED, the thickened area in the synthesized RPE-melanin thickness map correlated significantly with the PED volume, mirroring previous findings with original RPE-melanin thickness maps^12^. These results confirm that RPE changes in PED increase with PED size, demonstrating the utility of synthesized RPE-melanin OCT imaging for quantifying RPE activity, including intraretinal RPE migration, stacked RPE cells, and RPE dysmorphia.

In this study, the synthesized DOPU generated from standard OCT images was used for RPE-melanin OCT imaging in place of the DOPU derived from PS-OCT. The synthesized DOPU was calculated from single-polarization OCT intensity images, whereas the real DOPU requires two polarization components obtained via PS-OCT. Because the network input lacks complete polarization information, it likely relies on features in the intensity images that correlate with the DOPU, such as scattering properties^34^—for example, from melanin, a key source of DOPU contrast in retinal imaging^18^—and wavelength-dependent attenuation^35^. Although the exact relationship is not fully understood, these factors, which influence speckle formation, may allow the neural network model to approximate DOPU values^22^. The synthesized DOPU derived from these features closely resembled the actual DOPU and is considered suitable for substitution in the calculation of RPE-melanin OCT images.

Analysis of multimodal images, including synthesized RPE-melanin imaging, offers new perspectives on clinical AF findings. The similarity between the synthesized RPE-melanin thickness map and the NIR-AF image, along with the established role of the original RPE-melanin thickness map as an indicator of RPE-melanin change, suggests that synthesized RPE-melanin imaging provides a quantitative measure of RPE-associated melanin. Unlike NIR-AF, however, this synthesized imaging modality offers three-dimensional information about RPE-melanin. Both original and synthesized RPE-melanin B-scan OCT images revealed intraretinal RPE-melanin migration and accumulation at the RPE–Bruch’s membrane band in hyper NIR-AF lesions. The concurrent presence of hyper SW-AF in these lesions strongly suggests that these melanin-related findings originate from RPE changes such as intraretinal RPE migration, stacked RPE cells, and RPE dysmorphia^6,12^. These RPE changes are thought to reflect RPE activity^4^, indicating that synthesized RPE-melanin OCT imaging could be a valuable tool for evaluating RPE cellular activity in PEDs.

This study found a positive correlation between the PED volume and the area of active RPE lesions. Our prior research using PS-OCT and RPE-melanin OCT imaging, as well as a study on drusenoid PED, supports this correlation^4,6,12^. Several factors may contribute to RPE activation. Hypoxia, potentially resulting from reduced oxygen supply from the choriocapillaris, could play a role^36^. In larger PEDs, the increased distance from the choroid may hinder oxygen diffusion to the RPE^4^. Additionally, mechanical tension caused by architectural changes in the RPE band might be another contributing factor^12^. These mechanical forces could affect the extracellular matrix surrounding RPE cells, promoting RPE cell migration^37^. While the precise mechanism of RPE migration in serous PEDs remains unclear, both hypoxia and mechanical stress are possible causative factors in RPE changes.

In this study, the originalRPE_70_ area and synRPE_70_ area showed a strong correlation. However, the mean originalRPE_70_ area was significantly larger than the synRPE_70_ area. This difference stems from a systemic discrepancy between DOPU and synthesized DOPU values^22^, which is attributed to the limited performance of the neural network model used to generate the synthesized DOPU. Future improvements in the model’s architecture, learning hyperparameters, and choice of optimizer could potentially enhance its accuracy. Despite the difference in absolute values, the consistent correlation between the originalRPE_70_ and synRPE_70_ areas suggests that the deviation is systemic. Both measures are considered to reliably reflect the size of abnormal RPE lesions. Therefore, although the originalRPE_70_ and synRPE_70_ areas are not directly interchangeable, each can serve as a reasonable indicator of abnormal RPE area. In addition, it is conceivable that the gap between these values could be reduced by optimizing the F_RPE_ threshold. However, drawing firmer conclusions will require a larger number of cases.

This study had several limitations. First, the small sample size and lack of follow-up limited the scope of RPE change evaluation in serous PEDs. Larger, longitudinal studies are needed for a more comprehensive analysis. Second, this study focused on serous PED, a specific subtype of AMD, whereas AMD encompasses various conditions involving RPE changes^38^. Further investigation into these diverse conditions is necessary for broader clinical application of synthesized RPE-melanin OCT imaging. Third, the detection of intraretinal RPE changes in the present study—based on the presence of melanin and lipofuscin—may have been confounded by the possible presence of infiltrating inflammatory cells^39^. If inflammatory cells had ingested melanosomes and lipofuscin from disintegrating RPE cells, both RPE-melanin OCT and AF imaging might have misidentified these inflammatory cells as RPE cells. Additional histopathological studies are needed to address this limitation. Fourth, although a previous study showed a monotonic relationship between DOPU and melanin^17^, the measurement of RPE-melanin thickness may be influenced by factors such as the incident polarization state, the DOPU kernel size^18,20,28^, and the melanin packing density within RPE cells^40^. Therefore, while RPE-melanin thickness maps correlate proportionally with actual thickness, they should be interpreted with caution because they do not represent a direct measurement. Building on our previous research^12^, this study focused on regions where the RPE-melanin thickness exceeded 70 μm to investigate areas of RPE activity. However, a more detailed examination of the relationship between RPE-melanin thickness and RPE activity will require a larger study cohort and correlation with histopathological findings—important directions for future research. Fifth, for future clinical applications, it is important to generate synthesized RPE-melanin OCT imaging using data from conventional commercial OCT devices. In this study, synthesized RPE-melanin OCT imaging was generated from prototype multi-contrast PS-OCT measurement data to allow direct comparison with original RPE-melanin OCT imaging. A previous study demonstrated that the synthesized DOPU can be calculated from data acquired by standard commercial retinal OCT devices (DRI-OCT Triton; Topcon), which are capable of capturing both standard OCT and OCT angiography^22^. Because the attenuation coefficient can be calculated from standard OCT data^31^, this indicates the potential for directly generating synthesized RPE-melanin OCT imaging from commercial OCT data, warranting further investigation.

In conclusion, this study demonstrated the clinical utility of synthesized RPE-melanin OCT imaging for evaluating RPE changes in serous PEDs. Synthesized RPE-melanin OCT imaging enables three-dimensional assessment of findings typically observed in AF images. Importantly, it may be possible to generate synthesized RPE-melanin OCT imaging using conventional commercial OCT systems. This suggests that implementation could be achieved through software updates to existing commercial retinal OCT devices. Synthesized RPE-melanin OCT imaging is a promising tool and may serve as a next-generation imaging modality for the clinical evaluation of macular diseases.

## Electronic supplementary material

Below is the link to the electronic supplementary material.

Supplementary Material 1

## Data Availability

Data is provided within the manuscript or supplementary information files.
