# Supplementary material for: Evaluation of retinal pigment epithelium changes in serous pigment epithelial detachment using synthesized multi-contrast polarization-sensitive optical coherence tomography
Source: Sci Rep. 2025 Jul 7;15:24304. doi: 10.1038/s41598-025-09302-6 (PMC12234684; doi:10.1038/s41598-025-09302-6)
Supplement: Supplementary file 1 — Supplementary Material 1 [file 41598_2025_9302_MOESM1_ESM.pdf]

Evaluation of retinal pigment epithelium changes in serous pigment epithelial detachment using synthesized multi-contrast polarization-sensitive optical coherence tomography

Kosei Yanagida, Masahiro Miura, Hidetaka Noma, Toshihiro Mino, Shinnosuke Azuma, Thitiya Seesan, Shuichi Makita, Yoshiaki Yasuno

Supplementary Table

Mean original RPE-melanin thickness and mean synthesized RPE-melanin thickness.

Normal	original RPE-melanin thickness (μm)	synthesized RPE-melanin thickness (μm)
1	14.0	14.2
2	12.0	11.4
3	11.5	10.3
4	12.9	12.0
5	11.8	11.3
6	8.9	8.7
7	9.1	10.5
8	11.7	12.6
9	8.7	9.7
10	10.8	9.7
11	11.6	11.6
12	6.8	7.7
13	10.0	9.9
14	15.5	14.6
15	16.2	14.5
16	9.9	10.0
17	14.4	13.6
18	14.3	16.0
19	13.4	14.1
20	12.4	11.4
21	12.8	13.1
22	6.3	7.6
23	9.6	10.7
24	11.0	10.8
25	16.6	16.1
26	10.0	10.2
27	10.8	9.9

28	14.6	14.7
29	14.1	14.0
30	8.1	8.2
31	12.0	11.8
32	10.0	10.3
33	9.1	9.4
34	12.5	12.6
35	9.1	8.9
36	10.2	10.8

Serous PED	original RPE-melanin thickness (μm)	synthesized RPE-melanin thickness (μm)
1	20.9	19.6
2	16.5	18.0
3	18.2	14.1
4	17.6	16.6
5	16.8	20.9
6	13.7	11.2
7	13.0	13.3
8	15.6	18.1
9	20.3	21.3
10	9.1	11.6
11	12.2	15.0
12	15.4	14.8
13	14.6	16.2
14	17.3	19.0
15	20.2	19.9
16	14.4	13.2
17	14.5	14.6
18	14.7	14.9
19	18.2	18.8
20	18.1	15.3
21	13.3	13.4
22	15.0	16.3
